# Importance of basic life support training in rural India

**DOI:** 10.6026/973206300191011

**Published:** 2023-10-31

**Authors:** Durairaj Prakash, John Margaret, N. Siva Subramanian, Patel Vidhi Vishnu Bhai, Limbachiya Savankumar Navinbhai, Patel Shruti Ashvinbhai, Thakor Chhaya Arvindji

**Affiliations:** 1 Nootan College of Nursing, Sankalchand Patel University, Visnagar, Gujarat - 384315, India

**Keywords:** effect, basic life support training, village people

## Abstract

Basic life support (BLS) provided right away can lower fatality rates. Cardiac arrest typically results in death within minutes if it is untreated. Therefore, it is of interest to assess how BLS training affected villagers. The pre-experimental one-group pre-test post-test design was chosen for the investigation. A non-probability volunteer sampling technique was adopted to collect a sample of 220 village residents who met the inclusion requirements. The participants received training in basic life support that is achieved by using a real-life role model, hands-on CPR instruction. Checklist served as a standardized method for assessing the BLS training program. The pre-test and post-test's means were 23.05 and 56.51, respectively, and their respective standard deviations were 11.89 and 8.27. The 'z test' calculation result is 12.36) The results showed that BLS training was more successful for villagers and that they required regular BLS training programs to maintain their BLS skill level.

## Background:

One of the leading causes of death worldwide is cardiac disease. Emergency measures are required in cases of sudden cardiac arrest, which are happening more frequently in persons of all ages [1]. Treatments that can save a life are essential for avoiding deaths brought on by sudden cardiac arrest [2]. The first step towards regaining consciousness in cardiac arrest instances is a successful cardiopulmonary resuscitation. Lower cardiac arrest fatality rates are a result of health team members performing successful cardiopulmonary resuscitation at the scene [3]. Basic life support, a key component of the chain of survival decreases the arrest [4]. Theoretical understanding, however, is insufficient on its own to successfully carry out cardiopulmonary resuscitation. According to training and manual guidelines created for the health team, current knowledge should be updated, technical skills should be consolidated, and acceptable self-esteem related to the application should be formed. When someone has respiratory arrest, cardiac arrest, or airway blockage, BLS is the recommended course of action. It is a specific level of pre-hospital medical care provided by certified responders, including emergency medical technicians, to lessen the patient's critical status in the absence of advanced medical treatment. The BLS course trains students to identify a variety of life-threatening situations, do effective chest compressions, offer proper ventilations, and deliver an automated external defibrillator (AED) as quickly as feasible. BLS is a strategy used to save lives before getting to the hospital [5]. A hospital employee who works in healthcare must be sufficiently knowledgeable and informed of BLS and CPR [6]. If not treated right away, cardiac or cardiopulmonary arrest might result in serious morbidities or even death for the victim. The fundamentals of BLS include the early identification and CPR intervention of cardiac arrest patients. This enables the patient remain alive until the arrival of final medical care and the transfer to a hospital setting for additional advanced therapy. Using CPR, BLS aims to maintain an open airway, breathing, and circulation. Following a cardiac arrest, CPR is a life-saving technique used to regain cardiac and respiratory function [7]. It involves giving someone who is believed to be having a cardiac arrest a combination of external chest compression and mouth-to-mouth breathing [8]. Anyone who knows how to do it can perform it anywhere, at any time, without the need for tools or safety precautions. Precautions for patient transfer may include immobilization and splinting to prevent further injuries as well as cervical spine protection [9]. It instructs people of all ages on effective CPR techniques, how to operate an AED, and how to open a closed airway. According to AHA guidelines, the chance of survival after a cardiac arrest is reduced by 7%-10% for every minute defibrillation is delayed [10]. According to the European Resuscitation Council (ERC), early resuscitation and prompt defibrillation (within 1-2 minutes) can increase survival rates by more than 60% [11].The most recent BLS guidelines partially address issues with CPR quality that have been exposed in several articles in recent years, both inside and outside of hospitals [12]. Therefore, it is of interest to determine the level of awareness and attitudes about BLS among the locals in Kanza, Gujarat, in order to inform the development of the BLS program at this facility. We anticipate that all facets of BLS training for society will be improved and standardized as a result of this study.

## Methodology:

The study's research methodology was a one group pre-test post-test design. Using a non-probability volunteer sampling technique, 220 village residents who met the inclusion criteria were gathered. They were picked at random following verbal research information and their consent. Data were gathered using a set of pre-made questions. After giving a brief introduction to each villager and outlining the purpose of the study, a BLS demonstration was conducted. The researcher responded to the villagers' inquiries after the demonstration and exhorted everyone to do CPR. Performance was evaluated using a checklist, and the pre- and post-test scores were computed. The data was entered, analyzed, and conclusions were drawn using "Statistical Package for the Social Sciences (SPSS) version 22". Frequency and percentages were calculated for categorical variables in addition to mean and standard deviation for numerical variables. The efficiency of BLS training among villagers was evaluated using the Z test. Village people knowledge, attitude and practice on BLS falls under three categories (Table 1). Table 1show 75 % and above belongs to adequate level, 50-74% moderate level and below 49% is inadequate level. Table 2 shows Frequency and percentage distribution of samples based on age, occupation, religion, education, previous knowledge regarding BLS. Table 3 shows that mean of the pre-test and post-test was (23.05) and (56.51) and standard deviation of the pre-test and post-test was (11.89) and (8.27). The calculated 'z test' value (12.36) was greater than the table value (1.96) at 0.05 level.

## Results:

**Figure 1:**
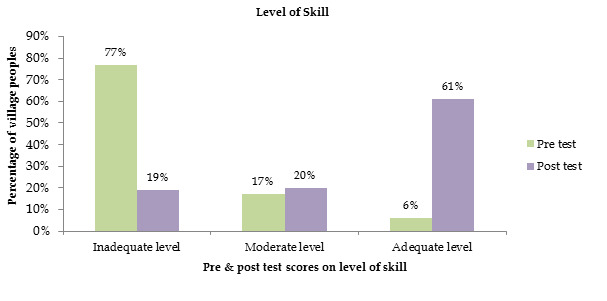
Frequency and percentage distribution of samples based on level of skill

Figure 1 shows frequency and percentage distribution of samples according to the knowledge score of health care personnel regarding biomedical waste management. It reveals that 77% of village peoples had inadequate, 17% of village peoples had moderate level, and 13 % of village peoples had adequate level in pre-test and 19% of village peoples had inadequate, 20% of village peoples had moderate level, and 61% of village peoples had adequate level in post-test.

## Discussion:

The study's objective was to assess BLS's impact on villagers. The results showed that BLS training was more successful for villagers and that they required regular BLS training programs to maintain their BLS skill level. According to research done by Kose on nursing students, basic life support training enhanced their understanding of and competency in basic life support procedures. Training in basic life support on a regular basis is crucial for nursing students to be competent in this area [13]. Roshana also did a study on medical staff, and it was shown that most of the staff members at her hospital are not adequately trained in CPR and BLS. These staff members' knowledge of CPR can be improved through training and experience. Thus, the hospital is advised to follow established CPR/BLS training and assessment standards [14]. In a research Chaudhary conducted with Nepalese healthcare professionals, he found that just 12% had adequate knowledge of basic life support, 55% had intermediate knowledge, and 32% had inadequate information. The survey demonstrated that the majority of participants lack adequate knowledge of basic life support among healthcare professionals. Significant correlations exist between the dependent and independent variables [15]. The goal of Alfakey study in Saudi Arabia was to evaluate students BLS knowledge, attitudes, and training status. She came to the conclusion that trained students had superior knowledge, skills, and attitudes to untrained students [16]. According to a study by Babar I in Pakistan, the general level of awareness among medical professionals is incredibly low, despite the fact that doctors have a greater understanding of BLS than dentists and nurses do [17]. The current study emphasizes the need for a BLS training course for healthcare professionals and other society members. According to a study by Juariah, basic life support is a collection of interventions used to restore and maintain the functioning of vital organs in victims of cardiac or respiratory arrest. Training can improve BLS practitioners' knowledge and abilities. This suggests that BLS instruction can improve adolescents' first aid knowledge and abilities in circumstances of cardiac or respiratory arrest [18]. According to a study conducted by Tadesse over half of the health science students at his facility lack the necessary BLS skills and knowledge. The study of anesthesia and medical departments, exposure to patients in need of basic life support, and training in basic and advanced life support were all closely associated to high knowledge. It is advised that exams and training be standardized to advance BLS expertise [19].

## Conclusion:

Data shows that there is a critical knowledge gap about BLS among rural and urban residents, as well as among hospital employees such as nurses, health assistants, auxiliary nursing midwives, and community medical assistants. This knowledge gap needs to be resolved in the future. Knowledge retention is influenced by BLS training and clinical exposure; all healthcare professionals should undergo some type of uniform training and evaluation. The purpose of the research study was to determine how well-informed the villagers were about BLS. Various recommendations, including a comparison study and a similar study to evaluate the effectiveness of programs to raise awareness of and share knowledge about BLS, might be made based on the study's findings. The results of the study supported the notion that BLS training programs for all healthcare professionals and members of society must be ongoing. Along with educational interventions, careful adherence to BLS recommendations and its oversight at all levels are also crucial.

## Figures and Tables

**Table 1:** Knowledge, Attitude and Practice level on BLS

**S. No**	**Knowledge level**	**Score**
1	Adequate level	75% and above
2	Moderate level	50 - 74%.
3	Inadequate level	Below 49%

**Table 2:** Frequency and percentage distribution of samples based on demographic variables

**S. No**	**Demographic variable**		**Experimental group**	
			**Frequency**	**Percentage**
1	Age (years)	20-30 years	66	30%
		31-40 years	71	32%
		41-50 years	51	23%
		Above 51 years	32	15%
2	Occupation	Sedentary worker	46	20%
		Moderate worker	98	45%
		Heavy worker	76	35%
3	Religion	Hindu	171	78%
		Muslim	38	17%
		Christian	11	5%
4	Education	Illiterate	36	16%
		Primary to high school	54	25%
		Higher secondary	63	29%
		Graduate	67	30%
5	Previous knowledge regarding BLS	Yes	61	28%
		No	159	72%

**Table 3:** Effect of BLS training among village peoples

**Group**	**Mean**	**Standard Deviation**	**Z Test**
Pre test	23.65	11.89	12.36 (Table value 1.96)
Post test	56.51	8.27	
